# Low Blood Levels of LRG1 Before Radical Prostatectomy Identify Patients with High Risk of Progression to Castration-resistant Prostate Cancer

**DOI:** 10.1016/j.euros.2022.09.002

**Published:** 2022-10-04

**Authors:** Ingrid Jenny Guldvik, Peder Rustøen Braadland, Shivanthe Sivanesan, Håkon Ramberg, Gitte Kristensen, Pierre Tennstedt, Andreas Røder, Thorsten Schlomm, Viktor Berge, Lars Magne Eri, Wolfgang Lilleby, Ian G. Mills, Kristin Austlid Taskén

**Affiliations:** a Department of Tumor Biology, Institute for Cancer Research, Oslo University Hospital, Oslo, Norway; b Institute of Clinical Medicine, University of Oslo, Oslo, Norway; c Norwegian PSC Research Center, Department of Transplantation Medicine, Institute of Clinical Medicine, University of Oslo, Oslo, Norway; d Department of Urology, Oslo University Hospital, Oslo, Norway; e Copenhagen Prostate Cancer Center, Department of Urology, Center for Cancer and Organ diseases, Copenhagen University Hospital-Rigshospitalet, Copenhagen, Denmark; f Martini-Klinik Prostate Cancer Centre, University Hospital Hamburg-Eppendorf, Hamburg, Germany; g Department of Clinical Medicine, University of Copenhagen, Copenhagen, Denmark; h Department of Urology, Charité Universitätsmedizin Berlin, Berlin, Germany; i Department of Oncology, Oslo University Hospital, Oslo, Norway; j Nuffield Department of Surgical Sciences, John Radcliffe Hospital, University of Oxford, Oxford, UK; k Department of Oncology, University of Cambridge, Addenbrooke’s Hospital, Cambridge, UK; l Cancer Research UK, Cambridge Research Institute, Li Ka Shing Centre, Cambridge, UK; m Patrick G. Johnston Centre for Cancer Research, Queen’s University of Belfast, Belfast, UK

**Keywords:** Biomarkers, Castration resistance, Hormone treatment, Noninvasive, LRG1, Prostate cancer, Radical prostatectomy, Surgery, Treatment resistance

## Abstract

**Background:**

After radical prostatectomy (RP), depending on stage, up to 40% of patients with prostate cancer (PCa) will experience biochemical failure (BF). Despite salvage therapy, approximately one-third of these patients will need permanent hormone therapy (pHT) and are at risk of progression to castration-resistant PCa (CRPC). Prognostic markers herald the need for neoadjuvant, adjuvant, or multimodal treatment.

**Objective:**

To evaluate the added value of blood LRG1 in predicting treatment failure in patients who have undergone radical prostatectomy (RP).

**Design, setting, and participants:**

We quantified LRG1 in serum or plasma sampled before radical prostatectomy from patients from the Martini-Klinik (Martini; *n* = 423), the Danish CuPCa cohort (CuPCa; *n* = 182), and Oslo University Hospital (OUH; *n* = 145).

**Outcome measurements and statistical analysis:**

The endpoints were BF, pHT, and CRPC. The association between LRG1 and survival outcomes was evaluated using Kaplan-Meier estimation and Cox proportional-hazards modeling. The added predictive value of LRG1 in nested models was estimated using the concordance index, time-dependent area under the receiver operating characteristic curve, and decision curve analysis.

**Results and limitations:**

In multivariable Cox models using preoperative characteristics, LRG1 was associated with an estimated lower risk of BF in the Martini cohort (adjusted hazard ratio [aHR] 0.68, 95% confidence interval [CI] 0.52–0.90) and in the CuPCa cohort (aHR 0.47, 95% CI 0.30–0.73). Using preoperative prognostic variables, our data showed that doubling of LRG1 was also associated with a lower risk of pHT receipt in the CuPCa cohort (aHR 0.43, 95% CI 0.20–0.93) and of CRPC development in the OUH cohort (aHR 0.32, 95% CI 0.15–0.69). Similar aHR values were observed using either preoperative or postoperative variables for all endpoints.

**Conclusions:**

PCa patients with high blood LRG1 are at lower risk of BF, pHT receipt, and progression to CRPC. Since LRG1 adds value to established prognostic models, new prognostic factor combinations including LRG1 should be considered in future studies.

**Patient summary:**

We measured concentrations of the blood-based protein LRG1 before surgery for prostate cancer. Patients with high LRG1 levels had better disease-free survival, suggesting that LRG1 can help in predicting prognosis.

## Take Home Message

Measurement of LRG1 in blood before radical prostatectomy may provide guidance on follow-up regimens and treatment decisions.

## 1 Introduction

Prostate cancer (PCa) leads the cancer statistics for men worldwide, ranking second in the latest GLOBOCAN report [1]. Most men are diagnosed at an early stage and are cured by radical prostatectomy (RP), although adjuvant multimodal management with hormonal therapy might be required in cases with adverse pathology [2]. The effect of hormone therapy (HT) commonly wanes within few years, and castration-resistant prostate cancer (CRPC) emerges. Several novel drugs have been launched and implemented for CRPC treatment [2], [3]. Recent trials have illustrated the delayed emergence of CRPC and prolonged survival if chemotherapy and second-generation antiandrogens are combined with standard HT upfront [4], [5].

Several blood-based biomarker panels have been proposed to improve the precision of predicting clinically significant PCa and adverse pathology in localized PCa [6], [7], [8], [9], [10], [11]. More recently, combining clinical information with gene expression tests has shown promising results, but the evidence level is not yet sufficient for the tests to be embedded in routine clinical practice [12].

In the treatment decision setting, immediate identification of patients who have a high risk of hormone resistance while on HT would be useful in addressing undertreatment for RP patients. Tests before RP that predict a disease course leading to CRPC are lacking. The use of noninvasive biomarkers before primary treatment may add clinical value to genomic tests in making better treatment choices and maximizing remission for each patient [3].

We previously identified the blood-based protein LRG1 as a noninvasive biomarker of aggressive and lethal PCa [13]. LRG1 is a secreted protein belonging to the TGF-β superfamily that affects a plethora of normal and pathological processes involved in cancer and immunity (recently reviewed by Camilli and colleagues [14]). The aim of our study was to evaluate the ability of LRG1 measurement to predict an advanced disease course and the development of CRPC in patients undergoing RP for localized PCa.

## 2 Patients and methods

### 2.1 Patient cohorts

**Fig. 1:**
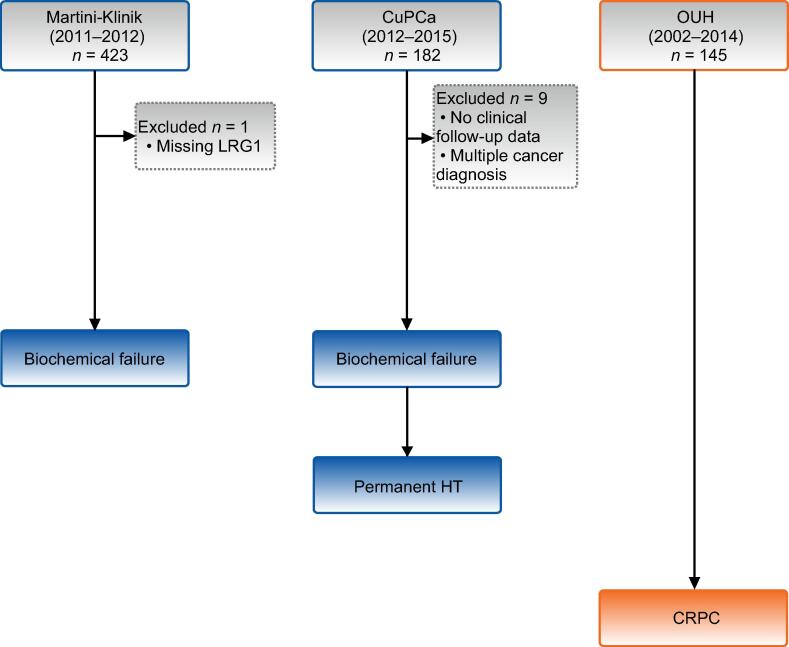
Study outline. LRG1 levels were determined in preoperative blood samples from men undergoing radical prostatectomy in three independent institutions. Propensity score matching of postoperative characteristics was used to generate a 1:2 case-control cohort of 423 serum samples from patients operated on at the Martini-Klinik (Martini). The endpoint for the Martini cohort was biochemical failure. Next, LRG1 in plasma from patients with postoperative high-risk, locally advanced disease or lymph node metastasis included in the Copenhagen uPAR prostate cancer (CuPCa) study was analyzed using biochemical failure and the start of permanent hormone therapy (pHT) as endpoints. Lastly, LRG1 was measured in 145 serum samples from the Prostate Biobank at Oslo University Hospital (OUH) enriched for patients starting permanent HT on prostate cancer recurrence. The primary endpoint for the OUH cohort was initiation of medication approved for castrate-resistant prostate cancer (CRPC). Figure created with BioRender.com.

This study includes individual patient data from three independent study cohorts. The overall study outline is depicted in Figure 1. Individual descriptions of study cohorts are provided below, and clinicopathological characteristics are listed in Table 1.

**Table 1:** Clinicopathological characteristics of the study cohorts

	Martini	CuPCa	OUH
Patients (*n*)	422	173	145
Year of surgery	2010–2012	2012–2015	2002–2014
Median follow-up, mo (interquartile range)	31 (25–37)	83 (74–92)	146 (127–171)
Median LRG1, μg/ml (interquartile range)	68 (40–88)	58 (45–72)	57 (45–87)
Median age, yr (interquartile range)	66 (61–70)	66 (62–69)	63 (59–67)
European Association of Urology risk group, *n* (%)			
Low	101 (23.9)	5 (2.9)	20 (13.8)
Intermediate	233 (55.2)	41 (23.7)	69 (47.6)
High	88 (20.9)	127 (73.4)	56 (38.6)
Median prostate-specific antigen, ng/ml (interquartile range)	7.4 (5.0–10.5)	9.5 (6.8–15.0)	9.4 (7.1–14.1)
Prostate-specific antigen category, *n* (%)			
<10 ng/ml	297 (70.4)	91 (52.6)	77 (53.1)
10–20 ng/ml	105 (24.9)	54 (31.2)	15 (10.3)
≥20 ng/ml	20 (4.7)	28 (16.2)	53 (36.6)
Pathological Gleason score, *n* (%)			
3 + 3	16 (3.8)	12 (6.9)	11 (7.6)
3 + 4	264 (62.6)	75 (43.4)	30 (20.7)
4 + 3	116 (27.5)	50 (28.9)	65 (44.8)
4 + 4	8 (1.9)	13 (7.5)	26 (17.9)
≥4 + 5	18 (4.3)	23 (13.3)	13 (9.0)
pT stage, *n* (%)			
T2a–b	22 (5.2)	4 (2.3)	14 (9.7)
T2c	221 (52.4)	62 (35.8)	48 (33.1)
T3a	127 (30.1)	65 (37.6)	46 (31.7)
T3b	52 (12.3)	42 (24.3)	37 (25.5)
Positive surgical margins, *n* (%)	78 (18.5)	52 (30.1)	55 (37.9)
Lymph node invasion, *n* (%)	31 (7.3)	21 (12.1)	6 (4.1)
Unknown	46 (10.9)	29 (16.8)	112 (77.2)
CAPRA-S score, *n* (%)			
0–2	167 (39.6)	16 (9.2)	24 (16.6)
3–5	189 (44.8)	80 (46.2)	58 (40.0)
≥6	66 (15.6)	77 (44.5)	63 (43.4)

CAPRA-S = Cancer of the Prostate Risk Assessment-Surgery nomogram; CuPCa = Copenhagen uPAR prostate cancer study; Martini = Martini-Klinik; ; OUH = Oslo University Hospital.

#### 2.1.1 Martini cohort

The Martini cohort comprised 423 patients whose blood was sampled in conjunction with RP at the Eppendorf Martini-Klinik (Hamburg, Germany) between 2010 and 2012. In October 2015 we first selected 141 patients who experienced biochemical failure (BF) and performed propensity score matching of postoperative clinical characteristics at a 1:2 case-control ratio to identify 282 BF-free control subjects. BF was defined as two consecutive rises in prostate-specific antigen (PSA) ≥0.2 ng/ml. One sample failed laboratory analysis. The study was approved by the ethics committee of Martini-Klinik (protocol code PV3652, December 29, 2010) and Health Region South-East (protocol code 2015/2372, February 9, 2016).

#### 2.1.2 CuPCa cohort

A cohort of 182 patients undergoing RP at Rigshospitalet (Copenhagen, Denmark) between 2012 and 2015 with postoperative high-risk or locally advanced PCa was initially included to evaluate the association between LRG1 and clinically significant endpoints. Seven patients were excluded because of lack of clinical follow-up data and two patients because of multiple cancer diagnoses. A baseline plasma sample was drawn at enrollment for the Copenhagen uPAR prostate cancer (CuPCa) study [15]. Baseline clinical and pathological information was collected from medical records. We retrospectively collected clinical follow-up data from medical records, including BF, the start of salvage or adjuvant radiotherapy, permanent hormone therapy (pHT; nonsteroidal antiandrogen and/or luteinizing hormone–releasing hormone [LHRH] agonist), and all-cause mortality up to September 2021. The Danish National Committee approved the study on Biomedical Research Ethics for the Capital Region (approval H-4-2011-071, August 30, 2011).

#### 2.1.3 OUH cohort

To study long-term follow-up, we included 145 patients undergoing RP at Oslo University Hospital (OUH) who were enrolled in the Prostate Biobank between 2002 and 2014. A retrospective review of postoperative outcomes was performed to assess the need for pHT (bicalutamide or LHRH agonist) and initiation of approved medication for CRPC (enzalutamide, abiraterone acetate, and docetaxel). A final update of clinical records was performed in September 2021. The study was approved by the ethics committee of Health Region South-East (protocol code 2015/1556, October 19, 2015). All participants in this study gave written consent.

#### 2.1.4 CuPCa de novo M1 disease

Baseline plasma samples from 19 patients with de novo M1 disease were drawn from the CuPCa study to complement the RP cohort and assess the association between LRG1 and disease burden. The study was approved by the ethics committee of Health Region South-East (protocol code 2015/1556, October 19, 2015). All participants in this study gave written consent.

### 2.2 Laboratory analyses

#### 2.2.1 LRG1 measurement

In general, levels of LRG1 in serum/plasma were measured in duplicates using a commercial solid-phase sandwich enzyme-linked immunosorbent assay (ELISA) kit (IBL International, Hamburg, Germany; catalog no. 27769) according to the manufacturer’s instructions. LRG1 concentrations in samples were calculated using a four-parameter curve-fitting analysis for a standard curve. Samples from the Martini and OUH cohorts were analyzed at the Institute for Cancer Research (OUH, Oslo, Norway). Samples from the CuPCa cohort were analyzed at the Rigshospitalet Central Laboratory (Copenhagen, Denmark). The overall correlation between the two laboratories was *R*^2^ = 0.61 (*r* = 0.78, 95% confidence interval [CI] 0.29–0.95) according to Pearson correlation analysis. The ELISA was also tested for variance between sample types (plasma vs serum; *n* = 40). The correlation was *R*^2^ = 0.42 (*r* = 0.67, 95% CI 0.45–0.79) on Pearson analysis.

##### 2.2.1.1 ELISA analysis at OUH

Serum samples were equilibrated at room temperature for 30 min before serial dilution with EIA buffer (1:2500). Samples were left to equilibrate in EIA buffer for 30 min before loading into plate wells. The absorbance was read using a Perkin Elmer Wallac VICTOR2 plate reader. The average intra-assay coefficient of variability (CV) was 3.6 (95% CI 0.2–12.5) between duplicates.

##### 2.2.1.2 ELISA analysis at Rigshospitalet Central Laboratory

Plasma analyses were performed using a fully automated BEP 2000 robotic system (Siemens Healthineers). In brief, samples were brought to room temperature for 30 min before serial dilution with EIA buffer (1:3000). Samples were left in EIA buffer for approximately 30 min before loading into plate wells. The average intra-assay CV was 0.95 (95% CI 0.0–7.6) between duplicates.

### 2.3 Statistical analyses

All statistical analyses were performed in R v4.1.2. The European Association of Urology (EAU) guidelines [16] were used to categorize patients into risk groups. LRG1 concentrations were log_2_-transformed before statistical analyses. Univariable linear regression models with LRG1 (continuous variable) as the dependent variable were built to evaluate associations with clinicopathological variables. BF-free survival was calculated as the time from RP until the first of two consecutively rising PSA measurements ≥0.2 ng/ml, or censored on the last date PSA measurement was performed. Times to pHT or CRPC were calculated to the dates for initiation of pHT or CRPC medication, respectively. Patients were censored at the date of death or date of final follow-up. Overall median follow-up times for each cohort were calculated as the time to death or last follow-up.

Kaplan-Meier estimates of times from RP to event outcomes (BF in the Martini and CuPCa cohorts, pHT in the CuPCa cohort, and CRPC in OUH cohort) were performed to illustrate surviving proportions of patient groups stratified by high (above median) or low (below median) LRG1 levels.

To assess whether LRG1 adds any value to established clinical characteristics, two distinct multivariable Cox proportional-hazards (PH) models were fitted using: (1) preoperative clinical characteristics (PSA [continuous], dichotomized Gleason score [GS; ≥4 + 4], and dichotomized clinical T stage [cT ≥3]); and (2) postoperative clinical characteristics (PSA [continuous], dichotomized pathological GS (≥4 + 4), dichotomized pathological T stage [pT ≥3], and lymph node invasion). These two models were used to assess for the adjusted association of preoperative serum LRG1 levels with BF, pHT, and CRPC in multivariable Cox PH analyses.

Discrimination was evaluated in terms of Harrell’s concordance index (*C* index) with optimism correction using internal resampling (200 bootstrap repetitions) with the *rms::validate* and *rms::calibrate* tools for *rms* v6.3.0). CIs for the change in *C* index were generated by running 200 iterations for resampling validation of the basic models (clinical variables alone) and full models (clinical variables + LRG1) separately (with 200 bootstrap repetitions) and within each loop, calculating the difference in the optimism-adjusted *C* indices.

Time-dependent increments in the area under the receiver operating characteristic curve (AUC) were assessed using *dynpred* v0.1.2. The added clinical benefit at relevant risk thresholds and time horizons was visualized via decision curve analysis (DCA) in R [17] using *dcurves* v0.2.0.

## 3 Results

### 3.1 Low preoperative LRG1 was associated with high BF risk in patients undergoing RP

**Fig. 2:**
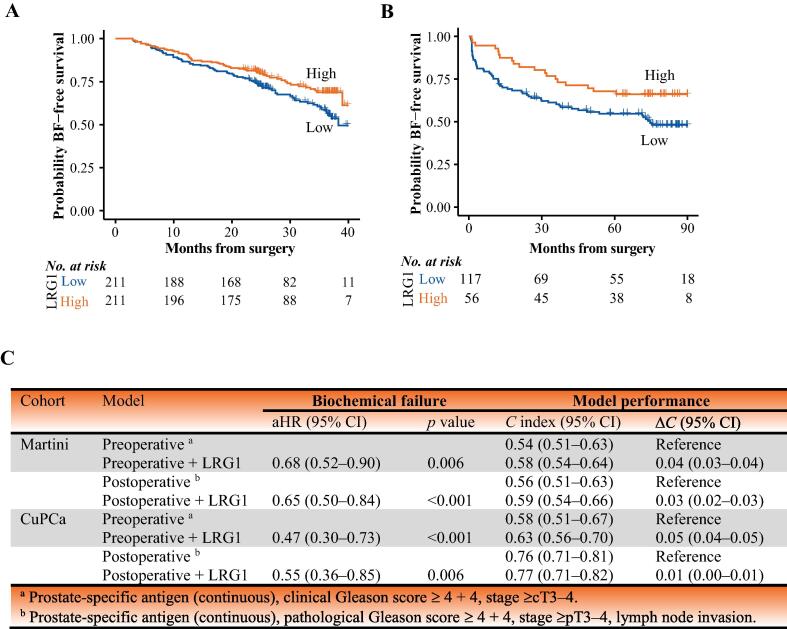
Kaplan-Meier biochemical failure (BF)-free survival among patients undergoing radical prostatectomy stratified by preoperative LRG1 level for (A) the Martini and (B) the CuPCa cohort. In both cases the median for the Martini cohort was used as the stratification cutpoint for low versus high LRG1. (C) Summary of multivariable Cox proportional-hazards preoperative and postoperative clinical models: adjusted hazard ratio (aHR) for BF after inclusion of LRG1 in the models, with model performance in terms of the concordance index (*C* index) for the Martini cohort (422 patients, 141 events) and CuPCa cohort (173 patients, 78 events). CI = confidence interval; ΔC = change in C index.

LRG1 was measured in serum collected before RP in 422 patients from the Martini cohort (Table 1). In this cohort, the median follow-up for patients who did not develop BF was 31 mo (interquartile range [IQR] 25–37). The median LRG1 concentration was 68 μg/ml (IQR 40–88). The risk of BF was higher for patients with LRG1 below the median (Fig. 2A). Linear regression confirmed an association between LRG1 levels and BF, extraprostatic extension, and lymph node invasion (Supplementary Table 1). We next evaluated the value of adding LRG1 to established preoperative and postoperative prognostic models using multivariable Cox PH modeling (Fig. 2C). When conditioned on these risk factors, a doubling in LRG1 in blood was associated with an estimated 32% and 35% lower risk of BF in preoperative and postoperative models, respectively.

In comparison to the clinical models, LRG1 addition led to an increment in discrimination (Fig. 2C). Among the time horizons, the increment in classification accuracy (AUC) with the full compared to the basic preoperative and postoperative models was greatest at 24 mo, which was also the time point by which most of the patients had developed BF (Fig. 2A, Supplementary Fig. 1A).

For both the preoperative and postoperative models, a greater net benefit was obtained on addition of LRG1 to the simple models for patients with a predicted BF risk threshold of 20% within 2 yr (Supplementary Fig. 1C).

As in the Martini cohort, the time to BF in the CuPCa cohort, which overall represented a more aggressive PCa population (Table 1), was shorter for the LRG1-low group than for the LRG1-high group (Fig. 2B), which was corroborated by an association with higher EAU risk group (Supplementary Table 2). In the CuPCa cohort, 78 men experienced BF and the median follow-up for those censored was 83 mo (IQR 74–92). Addition of LRG1 to the models improved discrimination (Fig. 2C), most notably in the first year (Supplementary Fig. 1B). Doubling of LRG1 was associated with 53% and 45% lower risk of BF according to the preoperative and postoperative models, respectively, conditioned on clinical variables.

For both the preoperative and postoperative models, the net benefit was greater for the full models than for the simple model, with a predicted risk of BF at 1 yr within the appropriate risk threshold interval of 7–20% in the CuPCa cohort (Supplementary Fig. 1D). At the 24-mo time horizon, the full models appeared to be well internally calibrated in the region in which most of the predicted probabilities were for both the Martini-Klinik and CuPCa cohorts (Supplementary Fig. 2).

We previously identified elevated LRG1 levels in patients with metastasis that we defined as either lymph node or distant metastasis. By extending the well-annotated CuPCa cohort to include patients with de novo distant metastasis, we were able to show lower LRG1 levels among patients with one positive lymph node (pN1) versus pN0 disease, and elevated LRG1 among patients with M1 disease compared to either pN0 or pN1 (Supplementary Fig. 3).

### 3.2 Low LRG1 predicted shorter time to postoperative pHT

**Fig. 3:**
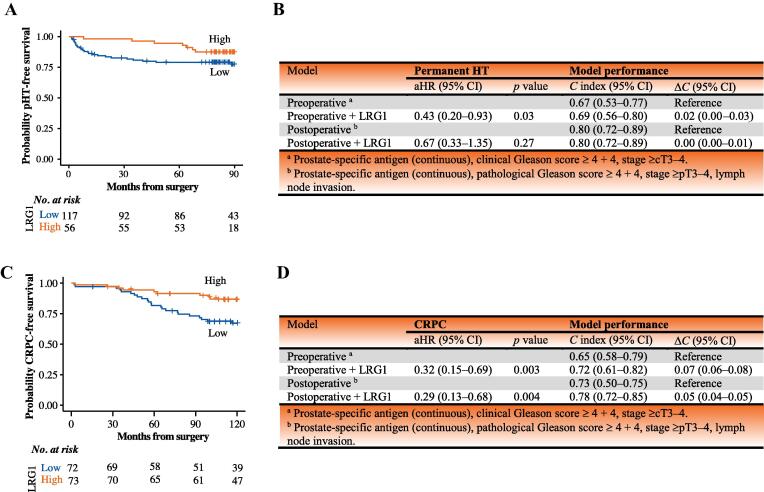
(A) Kaplan-Meier survival curves for the time from surgery to permanent HT in the CuPCa cohort stratified by preoperative plasma LRG1. (B) Summary of multivariable Cox proportional-hazards preoperative and postoperative clinical models: aHR for perm.HT receipt after inclusion of LRG1 in the models, with model performance in terms of the concordance index (*C* index) in the CuPCa cohort. (C) Kaplan-Meier survival curves for the time from surgery to CRPC in patients stratified by preoperative serum LRG1 in the OUH cohort. (D) Summary of multivariable Cox proportional-hazards preoperative and postoperative clinical models: aHR for CRPC after inclusion of LRG1 in the models, with model performance in terms of the *C* index in the OUH cohort. In all cases, the median for the Martini cohort was used as the cutpoint for low versus high LRG1. aHR = adjusted hazard ratio; CRPC = castration-resistant prostate cancer; pHT = permanent hormone therapy

A total of 168 patients were followed for >60 mo in the CuPCa cohort, of whom 27 started pHT within the 5-yr period. In multivariable Cox models adjusted for PSA, T stage, and Gleason grade, doubling of LRG1 was associated with a 57% lower risk of starting pHT (Fig. 3B). Addition of LRG1 to preoperative risk parameters improved discrimination (Δ*C* index 0.02), with an optimism-corrected *C* index of 0.67.

We observed an improvement in predicting the need for pHT when LRG1 was added to the established preoperative model for a risk threshold of 5–20%. Thus, LRG1 measurement can identify patients who may benefit from frequent monitoring and early intervention. There was also a minor improvement in predicting the need for pHT when LRG1 was added to the postoperative model (Supplementary Fig. 4A), albeit with a limited net benefit in this context (Supplementary Fig. 4B).

### 3.3 Patients who developed CRPC had low LRG1 at the time of surgery

CRPC developed in 16 of the CuPCa patients during follow-up. Early prediction of emerging resistance to HT is important, which prompted us to investigate whether LRG1 is associated with a lower risk of progression on HT. To this end, we analyzed LRG1 in serum from 145 patients in the OUH cohort. In total, 111 patients survived for a median of 146 mo (IQR 127–171) without starting therapy for CRPC. Patients starting on CRPC therapy had lower LRG1 levels than patients without CRPC (Fig. 3C and Supplementary Table 3). When modeled together with either preoperative or postoperative risk parameters in a Cox model, elevated LRG1 was associated with a considerable reduction in the risk of CRPC (Fig. 3D). Addition of LRG1 improved the prediction performance of both the preoperative and the postoperative model, in particular for early events (Supplementary Fig. 4C). Decision curve analyses indicated that addition of LRG1 to the models improved the net benefit at a threshold as low as 15% (Supplementary Fig. 4D) when predicting the need for CRPC medication within 5 yr.

## 4 Discussion

Our study demonstrates that LRG1 as a noninvasive biomarker adds prognostic information to preoperative and postoperative models assessing the risk of BF, initial need for pHT, and early progression to CRPC. To the best of our knowledge, LRG1 is the first preoperative blood-based biomarker associated with progression to CRPC for men undergoing RP. The main strength of our study is the use of well-annotated independent prostatectomy cohorts for assessment clinically important landmarks and long-term outcomes.

Surgery is followed by wound healing, a complex and fine-tuned inflammatory process in which LRG1 is involved in initiating a beneficial inflammatory response [18]. Low LRG1 levels impair resolution of inflammation, and the resulting high level of proinflammatory activity may potentiate survival of disseminated cancer cells in the proximity of surgical margins [18], [19].

LRG1 is also a marker of high levels of endothelial venules [20], specialized postcapillary venules that recruit naïve lymphocytes to lymph nodes [21]. Low LRG1 levels may reduce the number of T cells trained in lymph nodes to target cancer cells and thereby the antitumor immune activity [22].

In contrast to the present study, we previously showed that LRG1 predicted poor prognosis [13]. Our two studies differ in several aspects. Whereas RP cohorts were the focus this time, our previous work addressed long-term outcomes for patients undergoing a range of primary treatments, of which RP accounted for 43% of the cases. It is noteworthy that metastasis in our previous study included both lymph node and distant metastasis, whereas this time we were able to discriminate between pN1 and M1 disease. This enabled us to determine that elevated LRG1 was associated with de novo M1 disease, whereas low LRG1 was associated with dissemination to lymph nodes in patients with locally advanced disease. This observation supports the fact that there is a distinct difference in disease phenotype between patients with disseminated disease to the lymph nodes and early BF, and patients with distant metastasis.

Our study has some limitations. First, we used an exploratory design with retrospectively collected study cohorts, migrating from a predominantly intermediate-risk cohort to high-risk patients, so the study was underpowered for building and testing a well-calibrated prediction model. However, the models that included LRG1 that were built for the separate cohorts were well calibrated individually. Thus, validation of the models in independent cohorts is needed. Nevertheless, our results suggest that the predictive value of LRG1 might be generalizable heterogeneous RP cohorts. Building a prediction model in a larger study cohort and externally validating it in a sufficiently powered test cohort will be imperative for definitive conclusions. Second, HT was initiated for both adverse pathology and early BF, and although pHT was confirmed via medical record review, the analysis did not differentiate between initial adjuvant and salvage HT. In addition, decisions on the type of HT and its timing and duration were at the discretion of the treating physician, presumably on the basis of guidelines at that time. Third, there has inevitably been significant evolution of guidelines, standards of care, and patient populations from when the first patients were included in 2002 to the last in 2015. Furthermore, the patients in the cohorts were treated at three different institutions in three different countries, which also affected what was considered the standard of care. Finally, we have to assume that patient comorbidities also affected treatment decisions, and lack of comorbidity data meant that we could not adjust for comorbidity in our data. Such characteristics may have affected clinical decision-making, which should be considered when interpreting the results.

The endorsement of magnetic resonance imaging–guided biopsy and prostate-specific membrane antigen positron emission tomography in guidelines will lead to better diagnostic accuracy for patients undergoing RP. Consequently, there is a need to assess LRG1 in this contemporary setting. Furthermore, molecular and genomic profiling of blood and tissue samples is increasingly being utilized, and a head-to-head comparison for LRG1 will be needed to assess the added value in this context.

While these are important aspects for future investigation, our study indicates that LRG1 measurement in blood samples from patients with PCa before surgery may help in stratification for more intensive postoperative follow-up and multimodal treatment. Multimodal therapy may well be beneficial for patients with low LRG1. Preoperative LRG1 levels did provide an increase in benefit for clinical risk prediction of BF at a generalized threshold of 10–20%, representing a large patient cohort. There is a considerable risk of overdetection in patients with recurrence later than 5 yr [23]; however, LRG1 provided an increase in net benefit in predicting BF as early as 2 yr. Furthermore, HT is predominantly administered in patients with adverse pathology or early BF according to guidelines [2]. LRG1 added a net benefit in predicting early administration of HT at a fairly low risk threshold, thus providing an opportunity for early planning of multimodal treatment regimes for patients at high risk of further progression to CRPC. Intriguingly, patients with high LRG1 appeared to show good prognosis; if validated, this would indicate an opportunity for more conservative management for patients otherwise considered at high risk. Furthermore, measurement of blood LRG1 is easy and cheap and provides information on extrinsic factors that could affect treatment responses. LRG1 would be complementary to information on intrinsic tumor factors provided by genomic tests, which are now endorsed by guidelines.

## 5 Conclusions

Low LRG1 levels predict adverse pathology and early BF after surgery, as well as a short time to pHT and progression to CRPC. LRG1 provides added predictive accuracy to established risk factors. Future studies of independent large RP cohorts and prospective biomarker-driven trials are needed to validate the results.

  ***Author contributions***: Ingrid Jenny Guldvik had full access to all the data in the study and takes responsibility for the integrity of the data and the accuracy of the data analysis.

*Study concept and design*: Guldvik, Sivanesan, Ramberg, Braadland, Mills, Taskén.

*Acquisition of data*: Guldvik, Sivanesan, Ramberg, Braadland, Kristensen, Tennsted, Røder, Schlomm, Berge, Eri.

*Analysis and interpretation of data*: Guldvik, Sivanesan, Ramberg, Braadland, Lilleby, Mills, Taskén.

*Drafting of the manuscript*: Guldvik, Sivanesan, Ramberg, Braadland, Mills, Taskén.

*Critical revision of the manuscript for important intellectual content*: Guldvik, Sivanesan, Ramberg, Braadland, Kristensen, Tennsted, Røder, Schlomm, Berge, Eri, Lilleby, Mills, Taskén.

*Statistical analysis*: Guldvik.

*Obtaining funding*: Guldvik, Mills, Taskén.

*Administrative, technical, or material support*: Tennsted, Kristensen, Røder, Schlomm, Berge, Eri, Mills, Taskén.

*Supervision*: Mills, Taskén.

*Other*: None.

  ***Financial disclosures:*** Ingrid Jenny Guldvik certifies that all conflicts of interest, including specific financial interests and relationships and affiliations relevant to the subject matter or materials discussed in the manuscript (eg, employment/affiliation, grants or funding, consultancies, honoraria, stock ownership or options, expert testimony, royalties, or patents filed, received, or pending), are the following: Ingrid Jenny Guldvik and Ian G. Mills are owners of a patent for prostate cancer markers and their uses. The remaining authors have nothing to disclose.

  ***Funding/Support and role of the sponsor*:** This work was supported by the South-Eastern Norway Regional Health Authority (grant no. 2018620), the Norwegian Research Council (grant no. 249027), and Oslo University Hospital.

  ***Acknowledgments*:** The authors would like to express their gratitude to all the study participants in the Martini cohort (Germany), the CuPCa study (Denmark), and the Prostate Biobank (OUH, Norway) cohort, Kari Julien and Olov Ögren for excellent technical support, and Cecilia Adele Dunne for careful review of the medical records of the OUH patients.
