# Supplementary material for: Low Blood Levels of LRG1 Before Radical Prostatectomy Identify Patients with High Risk of Progression to Castration-resistant Prostate Cancer
Source: Eur Urol Open Sci. 2022 Oct 4;45:68–75. doi: 10.1016/j.euros.2022.09.002 (PMC9637679; doi:10.1016/j.euros.2022.09.002)

Supplementary Figure 1. A) Dynamic incremental *AUC(t)*(accuracy) of adding LRG1 to established risk models (EAU or post-surgery clinical model (CMpost; PSA continuous, pathological Gleason score ≥4+4, EPE, LNI)) for predicting BF throughout study follow-up in Martini cohort. **B)** Dynamic incremental *AUC(t)*(accuracy) of adding LRG1 to risk models (pre-clinical model (CMpre; PSA continuous, biopsy Gleason score ≥4+4, cTstage≥3) or CMpost) for predicting BF throughout study follow-up in CuPCa cohort. **C)** Decision curve analysis for net benefit of adding LRG1 to established risk models (EAU or CMpost) for predicting BF within 2-year post-surgery in Martini cohort. **D)** Decision curve analysis for net benefit of adding LRG1 to established risk models (CMpre or CMpost) for predicting BF within 1-year post-surgery in CuPCa cohort.

Supplementary Figure 2. Calibration plots for post-surgery prediction models including LRG1 for BF-free survival at 24 months in **A)** Martini (mean error= 0.014, 0.9 quantile = 0.026) and **B)** CuPCa (mean error 0.021, 0.9 quantile 0.05) . Variables included in models: PSA, Gleason score ≥4+4, extracapsular extension (ECE), lymph node invasion and LRG1. Bootstrap with 200 resampling repetitions.

Supplementary Figure 3. LRG1 plasma levels across different disease stages in an extended CuPCa cohort. Prostatectomy patients with pelvic lymph node dissection performed (n=144) were grouped according to no evidence of LNI (pN0= 123), only 1 positive pelvic LN (pN1= 16), more than 1 positive pelvic LN (pN2+ = 5), compared to patient with establish *de novo* M1 disease (n= 19).

Supplementary Figure 4. A) Dynamic incremental *AUC(t)*(accuracy) of adding LRG1 to risk models (pre-clinical model (CMpre; PSA continuous, biopsy Gleason score ≥4+4, cTstage≥3), post-surgery clinical model (CMpost; PSA continuous, pathological Gleason score ≥4+4, EPE, LNI)) for predicting need of permanent HT throughout study follow-up in CuPCa cohort. **B)** Decision curve analysis for net benefit of adding LRG1 to risk models (CMpre or CMpost) for predicting need of permanent HT within 2 years post-surgery in CuPCa cohort. **C)** Dynamic incremental AUC(t)(accuracy) of adding LRG1 to risk models (CMpre or CMpost) for predicting CRPC throughout study follow-up in OUH cohort. **D)** Decision curve analysis for net benefit of adding LRG1 to risk models (CMpre or CMpost) for predicting CRPC within 5 years post-surgery in OUH cohort.

Supplementary Figure 1.

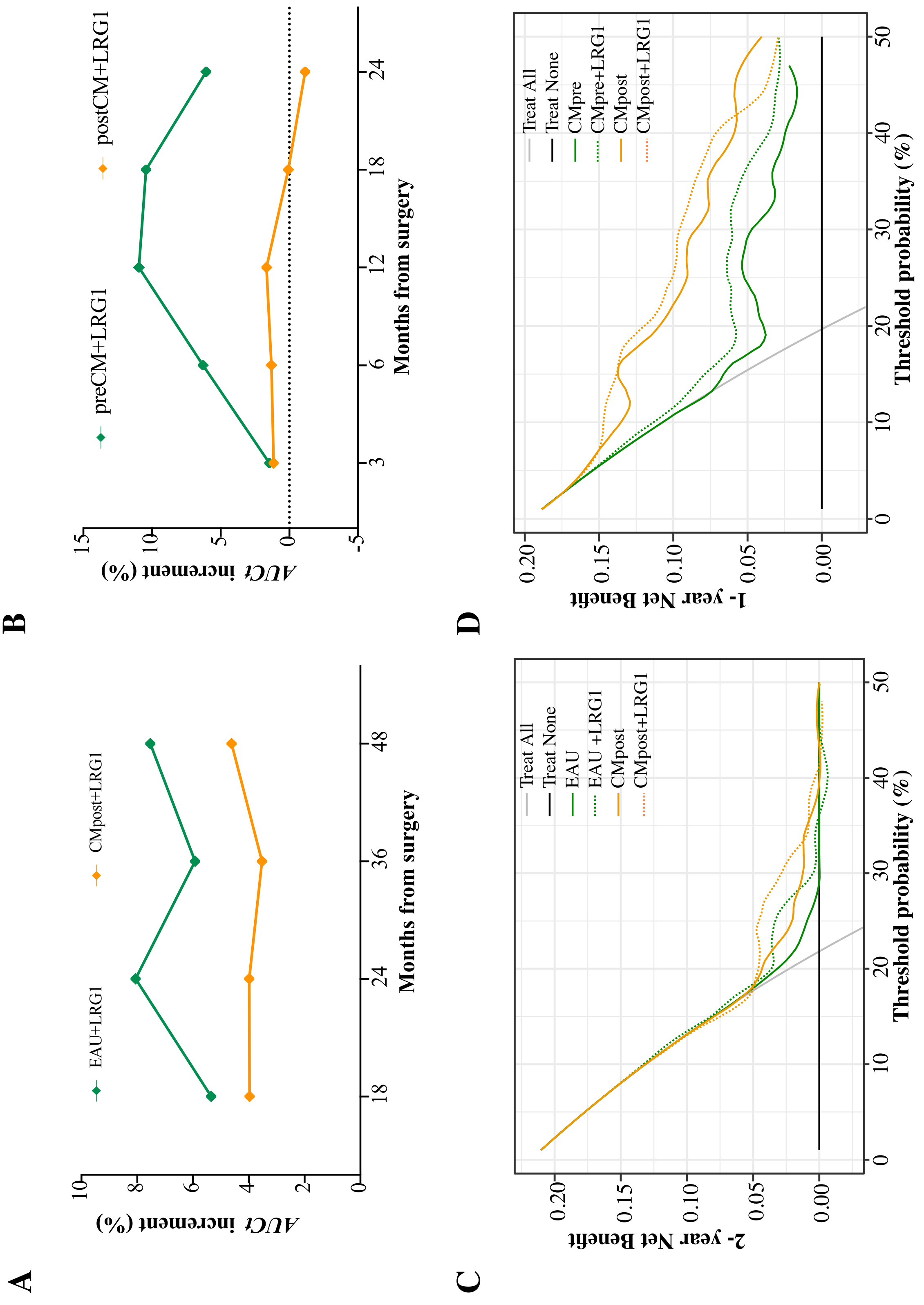

Supplementary Figure 2.

A

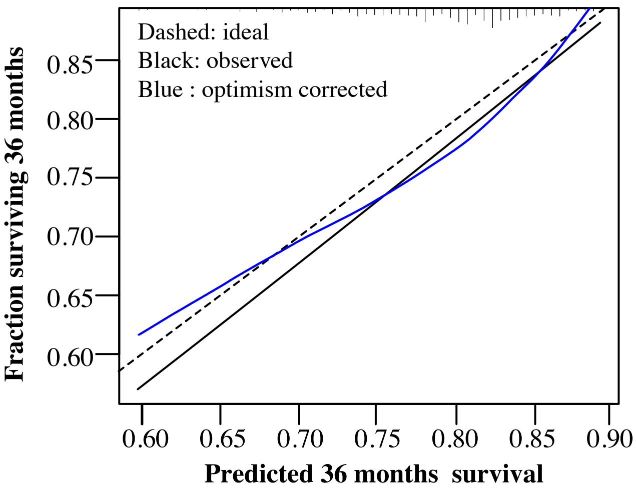

B

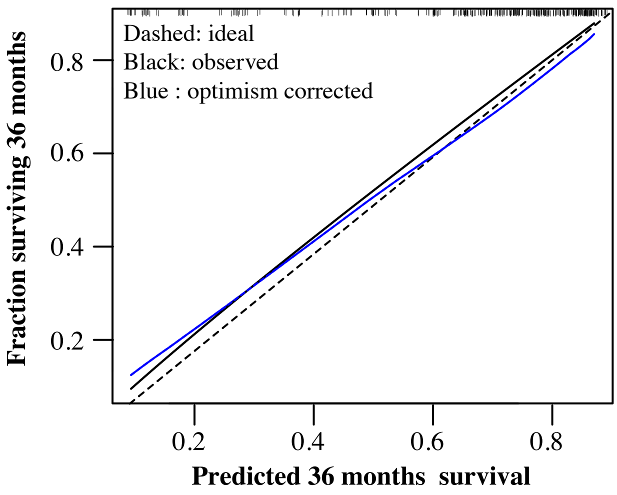

Supplementary Figure 3.

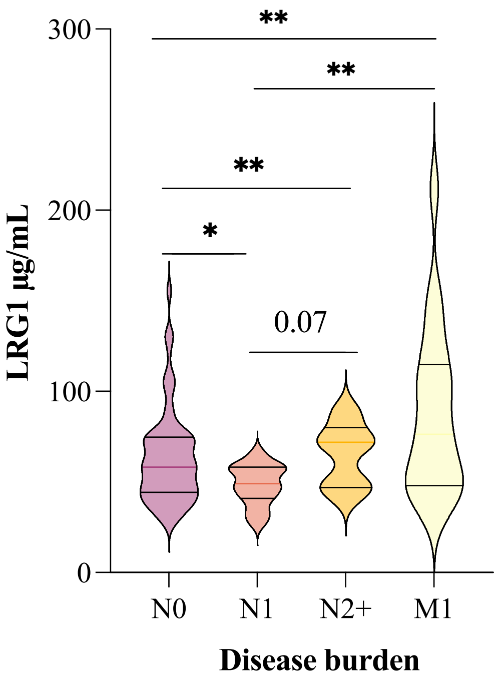

Supplementary Figure 4.

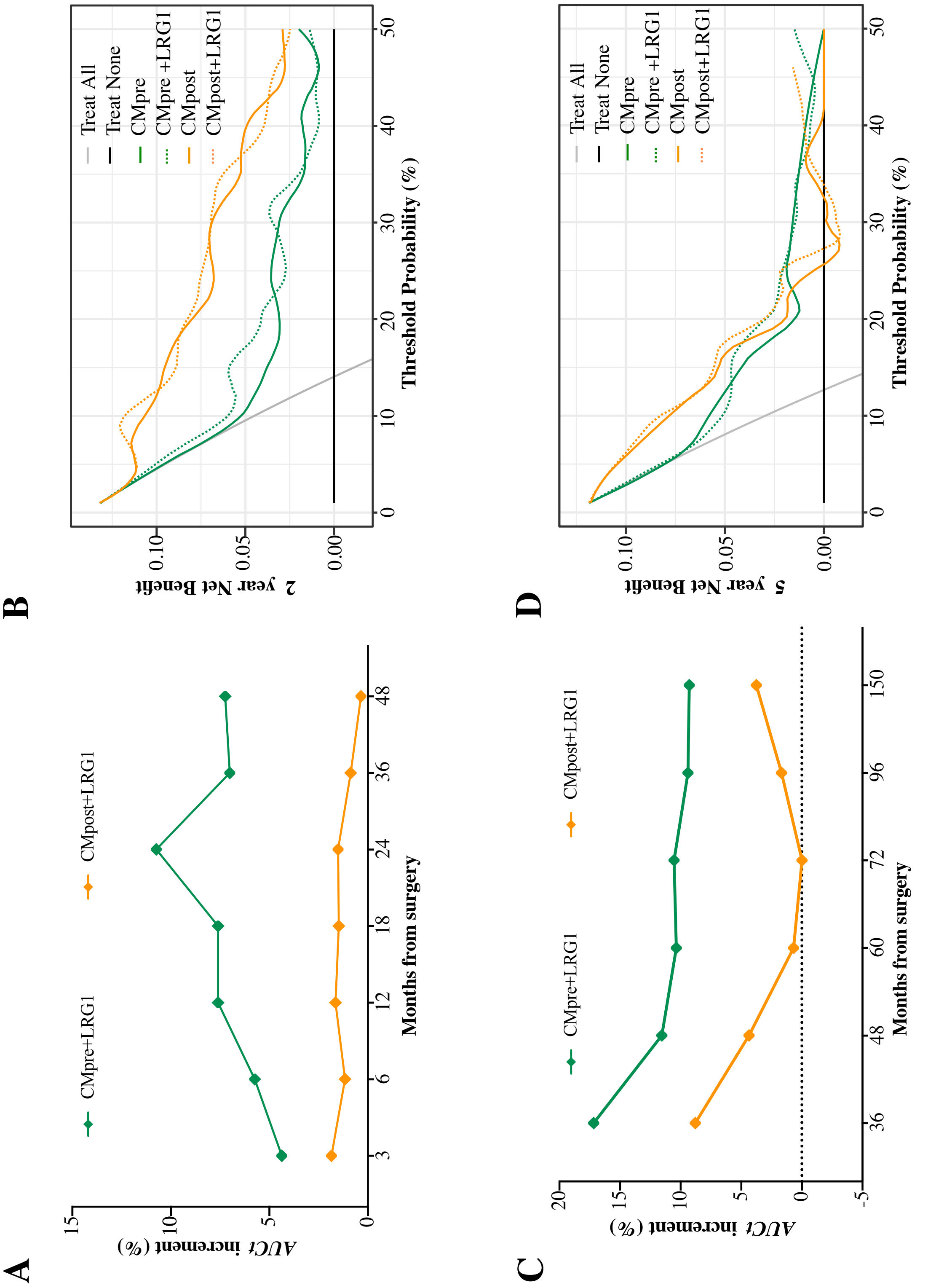

Supplement: Supplementary Figures [file mmc1.pdf]
