# Supplementary material for: Low Blood Levels of LRG1 Before Radical Prostatectomy Identify Patients with High Risk of Progression to Castration-resistant Prostate Cancer
Source: Eur Urol Open Sci. 2022 Oct 4;45:68–75. doi: 10.1016/j.euros.2022.09.002 (PMC9637679; doi:10.1016/j.euros.2022.09.002)
Supplement: Supplementary Tables [file mmc2.docx]

**Supplementary Tables**

**Table S1. Univariable linear regression of clinicopathological variables influencing LRG1 in Martini cohort. Ranking by *p-*value.**

**Table S2. Univariable linear regression of clinicopathological variables influencing LRG1 in CuPCa cohort. Ranking by *p*-value.**

**Table S3. Univariable linear regression of clinicopathological variables influencing LRG1 in OUH cohort.**

**Ranking by *p*-value.**

| **Table S1. Univariable linear regression of clinicopathological variables influencing LRG1 in Martini cohort. Ranking by *p-*value.** | | | | | |
| --- | --- | --- | --- | --- | --- |
|  | ***β*-coeff.** | | | **95% CI** | ***p*-value** |
| BF | -0.26 | | | (-0.50, -0.13) | <0.001 |
| EPE | -0.15 | | | (-0.28, -0.02) | 0.03 |
| LNI | -0.25 | | | (-0.51, -0.01) | 0.05 |
| SVI | -0.19 | | | (-0.39, 0.01) | 0.06 |
| PSA, ng/mL | -0.01 | | | (-0.02, 0.01) | 0.07 |
| PSM | -0.15 | | | (0.31, 0.02) | 0.09 |
| pGS | |  |  |  |  |
| 3+3 |  | | |  |  |
| 3+4 | -0.26 | | | (-0.59, 0.07) | 0.1 |
| 4+3 | -0.07 | | | (-0.36, 0.22) | 0.6 |
| 4+4 | 0.15 | | | (-0.19, 0.49) | 0.4 |
| ≥4+5 | 0.16 | | | (-0.09, 0.42) | 0.2 |
| EAU risk |  | | |  |  |
| Low | Ref. | | |  |  |
| Intermediate | 0.04 | | | (-0.10, 0.18) | 0.6 |
| High | 0.02 | | | (-0.13, 0.09) | 0.7 |
| BF = biochemical failure; EAU = European Association of Urology risk group; EPE = extra-prostatic extension; LNI = lymph node invasion; LRG1 = Leucine-rich alpha-2-glycoprotein 1; pGS = pathological Gleason Score; PSM = positive surgical margins; SVI = seminal vesicle invasion. | | | | | |

| **Table S2. Univariable linear regression of clinicopathological variables influencing LRG1 in CuPCa cohort. Ranking by *p*-value.** | | | | |
| --- | --- | --- | --- | --- |
|  | ***β*-coeff.** | **95% CI** | | ***p*-value** |
| EAU risk |  | |  |  |
| Low | Ref. |  | |  |
| Intermediate | -0.47 | (-0.83, -0.11) | | 0.01 |
| High | 0.33 | (0.08, 0.58) | | 0.01 |
| BF | -0.16 | | (-0.28, -0.03) | 0.02 |
| Age | 0.01 | | (0.00, 0.03) | 0.06 |
| CRPC | -0.18 | | (-0.39, 0.03) | 0.1 |
| Permanent HT | -0.13 | | (-0.30, 0.04) | 0.1 |
| PSM | -0.10 | | (-0.23, 0.04) | 0.2 |
| PSA, ng/mL | 0.00 | | (0.00, 0.01) | 0.3 |
| EPE | -0.06 | | (-0.19, 0.07) | 0.3 |
| LNI | -0.08 | | (-0.26, 0.11) | 0.4 |
| pGS |  | |  |  |
| 3+3 | Ref. | |  |  |
| 3+4 | -0.14 | | (-0.42, 0.13) | 0.3 |
| 4+3 | -0.13 | | (-0.37, 0.12) | 0.3 |
| 4+4 | 0.11 | | (-0.14, 0.36) | 0.4 |
| ≥4+5 | 0.07 | | (-0.13, 0.28) | 0.5 |
| SVI | 0.05 | | -0.10, 0.19 | 0.5 |
| BF=biochemical failure; coeff. = coeffiscient; CRPC= castrate-resistant prostate cancer; EAU= European Association of Urology risk group; EPE= extra-prostatic extension; HT= hormone therapy; LNI= lymph node invasion; LRG1=Leucine-rich alpha-2-glycoprotein 1; pGS=pathological Gleason Score; PSM=positive surgical margins; SVI=seminal vesicle invasion. | | | | |

| **Table S3. Univariable linear regression of clinicopathological variables influencing LRG1 in OUH cohort.**  **Ranking by *p*-value.** | | | |
| --- | --- | --- | --- |
|  | ***β*-coeff.** | **95% CI** | ***p*-value** |
| CRPC | -0.19 | (-0.32, -0.06) | 0.004 |
| PSA, ng/mL | -0.01 | (-0.02, 0.00) | 0.09 |
| BF | -0.09 | (-0.23, 0.05) | 0.2 |
| Permanent HT | -0.08 | (-0.26, 0.08) | 0.3 |
| Age | 0.01 | (-0.01, 0.02) | 0.4 |
| SVI | -0.06 | (-0.19, 0.07) | 0.4 |
| pGS |  |  |  |
| 3+3 | Ref. |  |  |
| 3+4 | 0.12 | (-0.14, 0.38) | 0.4 |
| 4+3 | 0.04 | (-0.19, 0.27) | 0.7 |
| 4+4 | 0.06 | (-0.14, 0.27) | 0.6 |
| ≥ 4+5 | 0.01 | (-0.14, 0.17) | 0.9 |
| EAU risk |  |  |  |
| Low | Ref. |  |  |
| Intermediate | 0.10 | (-0.07, 0.27) | 0.2 |
| High | -0.11 | (-0.25, 0.03) | 0.1 |
| EPE | -0.03 | (-0.20, 0.14) | 0.8 |
| PSM | -0.04 | (-0.21, 0.13) | 0.6 |
| BF=biochemical failure; coeff. = coefficient; CRPC= castrate-resistant prostate cancer; EAU= European Association of Urology risk group; EPE= extra-prostatic extension; HT= hormone therapy; LNI= lymph node invasion; LRG1=Leucine-rich alpha-2-glycoprotein 1; pGS=pathological Gleason Score; PSM=positive surgical margins; SVI=seminal vesicle invasion. | | | |
